# Kiwifruit effect on adipose tissue cell size and cholesteryl ester transfer protein gene expression in high-fat diet fed Golden Syrian hamsters

**Published:** 2019

**Authors:** Zahra Zaherijamil, Narjes Rezaei, Mohammad Hashemnia, Shirin Moradkhani, Massoud Saidijam, Iraj khodadadi, Ebrahim Abbasi Oshaghi, Heidar Tavilani

**Affiliations:** 1 *Department of Clinical Biochemistry, Hamadan University of Medical Sciences; Hamadan, Iran.*; 2 *Students Research Center, Hamadan University of Medical Sciences, Hamadan, Iran.*; 3 *Departments of Pathobiology, Veterinary Medicine Faculty, Razi University, Kermanshah, Iran.*; 4 *Medicinal Plants and Natural Products Research Center, Hamadan University of Medical Sciences, Hamadan, Iran.*; 5 *Research Center for Molecular Medicine, Hamadan University of Medical Sciences, Hamadan, Iran.*; 6 *Nutrition Health Research Center, Hamadan University of Medical Sciences, Hamadan, Iran.*

**Keywords:** Actinidia, Adipose tissue, Cholesteryl ester transfer protein, High fat diet

## Abstract

**Objective:**

The effects of kiwifruit on the histology and cell size of adipose tissue in hyperlipidemic models have not yet been reported. Therefore, this study aimed to investigate the effect of kiwifruit on the adipose tissue cell size and activity as well as the gene expression of cholesteryl ester transfer protein (CETP) in high-fat diet (HFD) fed hamsters.

**Materials and Methods:**

Forty-two male Syrian hamsters were divided into six groups. Control normal (CN) hamsters received normal diet, control HFD (CHF) were fed with a HFD plus a normal diet (15% butter fat + 0.05% cholesterol + a normal diet). Two groups were fed with normal diet including kiwifruit (1.86; Nd.1 or 3.73 g/kg; Nd.2) and two groups were fed with HFD including kiwifruit (1.86;HFd.1or 3.73 g/kg; HFd.2), for 8 weeks.

**Results:**

Histological examination of adipose tissue showed that the cell size was significantly reduced in the kiwifruit-treated groups (low and high dose) in comparison to their control groups (p<0.05). Kiwifruit supplementation (low and high dose) in normal and HFD groups significantly increased gene expression of CETP in adipose tissue. Kiwifruit had no significant effect on serum concentration of low-density lipoprotein cholesterol, total cholesterol and triglyceride. Although, high-density lipoprotein cholesterol concentration increased in HFD-fed hamsters supplemented with 3.73 g/kg of kiwifruit (p<0.05).

**Conclusion:**

Kiwifruit consumption reduces the size of adipocytes and increases the expression of CETP gene in adipose tissue cells. Despite the increases in CETP expression in adipose tissue, its activity in serum was not changed following kiwifruit supplementation.

## Introduction

Hyperlipidemia refers to an increase of blood lipid profile, which is also called dyslipidemia due to causing multiple disorders in the metabolism of lipoproteins. In this case, total cholesterol, low density lipoprotein cholesterol (LDL-C), and triglyceride increase while high density lipoprotein cholesterol (HDL) decreases (Nelson, 2013 ▶). Cholesterol-based diets are a common way to induce hypercholesterolemia in laboratory animals (Stancu et al., 2015 ▶).

In treatment with a high-fat diet, excess energy is stored in triglyceride form in adipose tissue cells. Obesity is characterized by an increase in fat mass, which is due to hyperplasia (i.e. increased the number of fat cells) or hypertrophy (i.e. increased fat cells size) (Hariri and Thibault, 2010 ▶). In this regard, hamster is a good animal to model the metabolism of cholesterol and lipids, and shows a similar reaction to changes in dietary lipids as humans (Kahlon et al., 1996 ▶).

Kiwifruit is considered a beneficial fruit with high levels of antioxidant compounds including vitamin C and E, polyphenols, and flavonoids (Shastri et al., 2012 ▶). One of the important effects of kiwifruit that has been considered in recent years is its beneficial effects on the lipid profiles (Gammon et al., 2013 ▶; Chang and Liu, 2009 ▶). Several studies have shown that kiwifruit has positive effects on HDL, however, the mechanism by which kiwifruit affects lipid profile and HDL-cholesterol levels is still unknown. Nevertheless, the effects of kiwifruit on the cell histology and size in adipose tissue in hyperlipidemic models, have not yet been studied. Changes in adipose tissue cells can lead to significant changes, including changes in the cholesteryl ester transfer protein (CETP) gene expression (Radeau et al., 1995 ▶).

CETP has an important role in lipid profile of individuals. In serum, CETP passes cholesterol ester from HDL to VLDL and LDL, resulting in a decrease in HDL-C levels (Barter et al., 2003 ▶). The CETP protein is synthesized by some tissues including liver, spleen, heart, skeletal muscle, and adipose tissue. Adipose tissue of hamster is the main site for CETP mRNA production (Jiang et al., 1991 ▶; Tall, 1993 ▶).

It was reported that diet with high content of fat and cholesterol, leads to increased activity of CETP (Stein et al., 1990 ▶; Son and Zilversmit, 1986 ▶). Various compounds affect CETP activity (Javandoost et al., 2018). It was reported that administration of apple polyphenols to hamsters led to reduction of CETP activity (Lam et al., 2008 ▶). It has also been shown that antioxidant compounds such as quercetin, isoliquiritigenin, and vitamin E are effective in inhibiting CETP activity in hamsters (Hirata et al., 2012 ▶; Shen et al., 1996 ▶). In this study, the presence of phenolic and flavonoid compounds (such as isoliquiritigenin and quercetin) in kiwifruit extract was confirmed. Because kiwifruit has high level of vitamin E, it is expected that it may inhibit CETP activity. The aim of this study was to investigate the effect of kiwifruit on the adipose tissue cell size and activity as well as cholesteryl ester transfer protein (CETP) gene expression in high-fat diet (HFD) fed hamsters.

## Materials and Methods

**Animals and diets**

In this experimental study, 42 eight-week-old Syrian hamsters with the mean weight of 120-150 g were obtained from the Animal Center of Hamadan University of Medical Sciences. Initially, the hamsters were left for 10 days for adaptation; light and darkness each for 12 hours and with the moisture recommended for laboratory animals and automatic ventilation. The ethical considerations of working with animals were also respected and the research was approved by Ethics committee of Hamadan University of Medical Sciences. 

To prepare HFD, standard dietary food was powdered and mixed with 15% butter and 0.05% cholesterol (Kahlon et al., 1996 ▶). After preparation and drying, it was used as a daily meal of HFD fed hamster. Control normal of hamsters received a standard chow diet.

Kiwifruit was purchased from local market, and after washing and cutting, it was completely mixed using a mixer. The amount of kiwifruit given hamsters, was calculated according to their weight, which was equivalent to taking 1 and 2 kiwifruit per day by a human being of 70 kg, according to human studies (1.86 or 3.73 g/kg, respectively) (Gammon, et al., 2013 ▶, Chang and Liu, 2009 ▶, Brevik et al., 2011 ▶). Each dose of kiwifruit (1.86 g/kg) was combined with 1 ml of water for ease of gavage; finally, kiwifruit was given to animals at two doses of 1.86 and 3.73 g/kg for 8 weeks.

The hamsters were categorized into six groups:

Control hamsters were fed with normal diet (ND) plus normal saline (CN).

Hamsters fed with normal diet (ND) plus 1.86 g/kg kiwifruit (Nd.1).

Hamsters fed with normal diet (ND) plus 3.73 g/kg kiwifruit (Nd.2).

Hamsters fed with high-fat diet (HFD) plus normal saline (CHF). 

Hamsters fed with high-fat diet (HFD) plus 1.86 g/kg kiwifruit (HFd.1).

Hamsters fed with high-fat diet (HFD) plus 3.73 g/kg kiwifruit (HFd.2). 

At the end of the study, the hamsters were anesthetized using chloroform steam, and sacrificed and the blood was taken from the lower inferior vein. Their serum was isolated and kept at -80°C for biochemical tests and measurements of enzyme activity. Different tissues including adipose tissue and liver were removed. After washing in the physiologic serum, some of them were placed in 4% formalin for histopathology examination, and the rest were transferred to -80 °C until analysis.

**Serum lipid profile**

The serum levels of cholesterol and triacylglycerol were measured by using enzymatic kit (Pars Azmun Company) based on photometric method. HDL-C and LDL-C levels were determined directly by commercial kits (Paad.CO kit), based on enzymatic reactions.

**CETP activity**

Activity of CETP in serum was determined by a ﬂuorometric kit (Abcam 196995) according to the manufacturer's recommendations. By CETP action, transfer of the fluorescent phospholipid to the acceptor molecule is done which increases the intensity of fluorescence light (Ex/Em=480/511 nm). CETP activity was calculated based on fluorometric intensity and expressed as pmol/μl/hr.

**CETP gene expression:**

Extraction of RNA from about 200 mg of epididymal adipose tissue was done using Accuzol solution (Bionner, Korea). The quality of the extracted RNA was checked by electrophoresis on the agarose gel. The corresponding cDNA was then prepared according to the Revert AidTM First Strand cDNA Synthesis kit instruction. Finally, the Real Time PCR reaction was done by Syber premix Ex kit protocol. The sequences of primers were as follows: 

for CETP: forward 5'- TCGACATCATCAACCCCGAG-3' and reverse 5’- GACGCTCCAGTTCCATTGTG-3', and for beta-actin used as housekeeping gene: forward 5'- GGG AAA TCG TGC GTG ACA TTA AG-3' and reverse 5'-TGT GTT GGC GTA CAG GTC TTT G-3'.

**Histological analysis of adipose tissue**

For histopathologic studies, adipose tissues were fixed in formalin 10% for 24 hours. In the next step, paraffin sections were made and sections with a thickness of 5 μm were prepared by rotating microtome. The sections were then stained with hematoxylin-eosin. Histopathologic slides were evaluated by an optical microscope. Digital images with 640×400 pixels and a magnification of X1000 were taken from tissues by a digital camera (Dino capture; version 1.2.7) attached to the microscope. To increase the contrast, the images were processed by Adobe Photoshop software. In this study, only normal and non-overlapping cells were examined and 50 cells per animal were evaluated. Morphometric image analysis was performed by ImageJ (version 1.47) software following the steps enumerated below: 

-Pictures were opened through the following menu: File>Open. 

-Brightness and contrast were adjusted from the following menu: Image>Adjust>Brightness/Contrast. 

-The noise of images was removed through the following menu: Option Process>Filters>Median.

-To increase the image quality and determine the cell border, the Kuwahara and Mexican hat filters from the Plugins panel were used. The Kuwahara filter reduces the noise while preserving the edge; and the Mexican Hat Filter helps to separate signal from the noise by applying Laplacian of Gaussian filter. 

-Adjustment of the measurement scale: A line was drawn between two points with a determined distance. ‘Set Scale’ was selected from the ‘Analyze Options’. The distance between the lines and its length were written in their respective fields, and ‘OK’ was clicked. 

-The cells boundary was specified by drawing a line around it by ‘Freehand line’. 

-Cell parameters, including the cell area, the perimeter, the diameter, and the circularity were measured through: Analyze>Set measurement>Measure. 

**Total phenolic content and total flavonoid content of kiwifruit**

Alcoholic and hydroalcoholic extracts were prepared from fresh and dried kiwifruit. The level of phenolic and flavonoid compounds of kiwifruit was determined according to a previous report (Abbasi Oshaghi et al., 2015 ▶). Total phenolic content was expressed as mg equivalents of gallic acid/gram extract. Total flavonoid content was also determined using AlCl_3_ and quercetin as standard. Total flavonoid content was presented per mg equivalents of quercetin/gram extract 

The analysis of pyrogallol and vitamin C was done using an HPLC system (WATERS Co. USA.), equipped with a C18 column (250 mm×4.6 mm i.d, 5 μm particle size), UV detector (210 nm), and isocratic pump. The mobile phase flow rate for pyrogallol (acetonitrile and water) and vitamin C (acetonitrile and 0.1% phosphoric acid) was 1 ml/min. 

**Statistical analysis**

In this study, SPSS-19 software was used to analyze the results. We used Kolmogorov Smirnov test to determine normality of data. For non-normal-distributed data, Kruskal-wallis and Mann-Whitney tests and for data with normal distribution, One-way ANOVA test with Tukey *post hoc* was used. Data are presented as mean±SD and the level of significant difference was considered p<0.05.

## Results

**Effect of kiwifruit consumption on serum lipid profile**

After 8 week treatment of hamsters with HFD, significant increases in total cholesterol (TC) (p<0.05), HDL-C (p<0.01), LDL-C (p<0.05) and triglyceride concentration (p<0.01) were detected in CHF group in comparison to control normal group. Kiwifruit had no significant effect on TG, LDL-C and TC levels in treated groups versus their controls (p>0.05).

HDL-C concentration increased in HFD-fed hamsters supplemented with dose 1 (1.86 g/kg) and 2 (3.73 g/kg) of kiwifruit and the change was significant following administration of kiwifruit 3.73 g/kg. HDL-C level in normal hamsters was not affected by kiwifruit supplementation. The data are shown in Table 1.

**Table 1:** Concentration of serum total cholesterol, triglyceride, low density lipoprotein-cholesterol (LDL-C), high density lipoprotein-cholesterol (HDL-C), in control and high-fat diet fed hamsters (n=7 in each group) supplemented with kiwifruit

**Parameter**	**CN**	**Nd.1**	**Nd.2**	**CHF**	**HFd.1**	**HFd.2**
**Cholesterol (mmol/L)**	2.1±0.38	1.7±0.38	1.79±0.37	7.2±3.2#	7±3.2	7.2±3.4
**Triglyceride (mmol/L)**	2.49±0.51	1.87±0.91	1.87±0.46	24.9±7.5 ###	15.2±6.7	18±7.9
**LDL-C (mmol/L)**	0.26±0.05	0.26±0.08	0.25±0.05	0.71±0.33#	0.63 ± 0.5	0.61±0.4
**HDL-C (mmol/L)**	0.63±0.11	0.64±0.13	0.64±0.11	0.94±0.2 ##	1.1±0.09	1.2±0.24**

The data are expressed as mean±SD. Mann-Whitney test was used for cholesterol, LDL-C and HDL-C. ANOVA test with Tukey *post hoc* was used for triglyceride.

Abbreviation: CN, Control normal; CHF, Control high fat; HFd.1, High-fat diet plus 1.86 g/kg kiwifruit; HFd.2, High-fat diet plus 3.73 g/kg kiwifruit; Nd.1, normal diet plus 1.86 g/kg kiwifruit; Nd.2, normal diet plus 3.73 g/kg kiwifruit.

** P<0.01 HFd2 versus CHF.

# p<0.05

## p<0.01

### p<0.001 CHF versus CN group.

**Effect of kiwifruit consumption on serum CETP activity**

**Figure 1:**
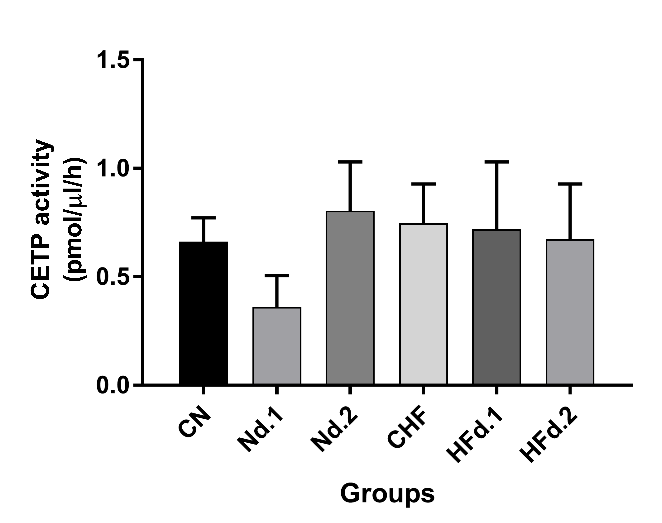
Activity of cholesteryl ester transfer protein (CETP) (pmol/µl/hr) in serum of hamsters (n=7 in each group). The data are expressed as mean±SD. Mann-Whitney test was used for statistical analysis

Abbreviation: CN, Control normal; CHF, Control high fat; HFd.1, High-fat diet plus 1.86 g/kg kiwifruit; HFd.2, High-fat diet plus 3.73 g/kg kiwifruit; Nd.1, normal diet plus 1.86 g/kg kiwifruit; Nd.2, normal diet plus 3.73 g/kg kiwifruit.

Our results showed that HFD and kiwifruit treatment had no significant effect on CETP activity (Figure1).

**Effect of kiwifruit consumption on adipose tissue CETP gene expression**

**Figure 2:**
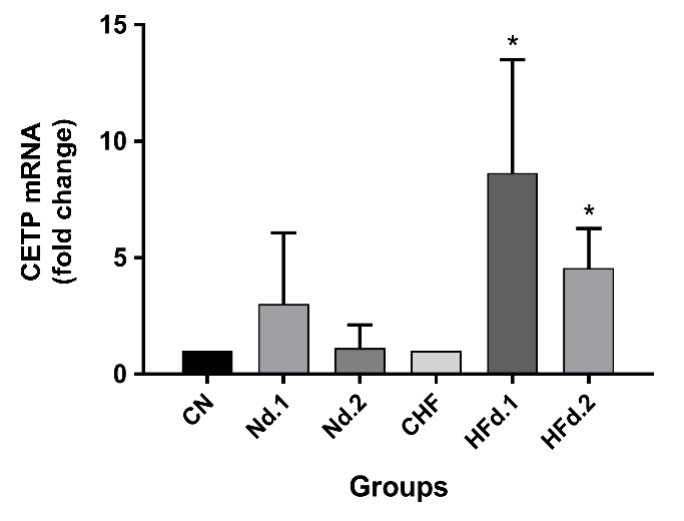
Gene expression of cholesteryl ester transfer protein in adipose tissue of hamsters (n=7 in each group). The data are expressed as mean±SD. ANOVA test with Tukey *post hoc* was used. *p<0.05 HF.treatment groups versus control

Abbreviation: CN, Control normal; CHF, Control high fat; HFd. 1, High-fat diet plus 1.86 g/kg kiwifruit; HFd.2, High-fat diet plus 3.73 g/kg kiwifruit; Nd.1, normal diet plus 1.86 g/kg kiwifruit; Nd.2, normal diet plus 3.73 g/kg kiwifruit.

As shown in Figure 2, after 8 weeks of kiwifruit consumption, CETP gene expression was increased significantly in HFD- fed groups in comparison to control groups (p*<*0*.*05). CETP expression also increased in normal diet groups but it was not significant.

**Histological analysis of adipose tissue**

**Figure 3:**
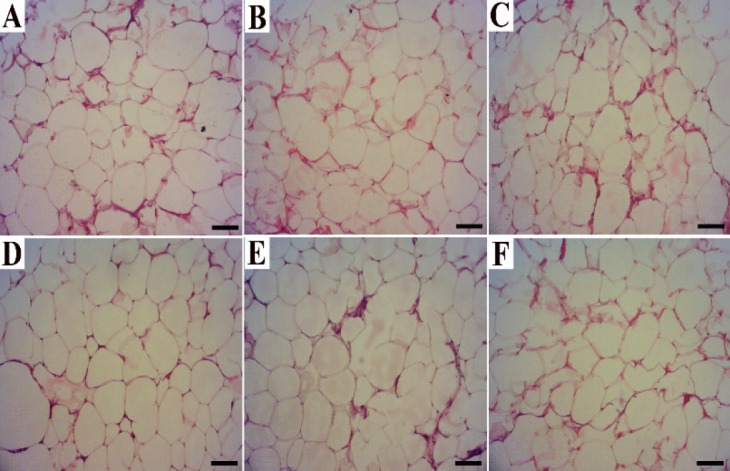
Histopathological observation in the white adipose tissues of the experimental rats (H&E, scale bar=100 μm). A) CN, Control normal; B) Nd.1, Normal diet with dose 1 (1.86 g/kg) of kiwifruit; C) Nd.2, Normal diet with dose 2 (3.73 g/kg) of kiwi fruit; D) CHF, Control high-fat group; E) HFd.1, High-fat diet plus 1.86 g/kg kiwifruit; F) HFd.2, High-fat diet plus 3.73 g/kg kiwifruit; Nd.1, normal diet plus 1.86 g/kg kiwifruit; Nd.2, normal diet plus 3.73 g/kg kiwifruit

In the microscopic examination of histopathologic slides of adipose tissue, no tissue abnormalities were observed and the structures were normal (Figure 3), but each of the cell size determinant parameters, including cell area, mean diameter, perimeter and circularity of cells, was significantly larger in HFD-treated animals than normal diet-treated hamsters (p<0.05). Also, the cell size parameters were significantly reduced in the kiwifruit-treated groups in comparison to the control groups (p<0.05) (Table 2).

**Table 2:** Comparison of morphometric parameters of fat cells in control and high-fat diet fed hamsters supplemented with kiwifruit (n=7 in each group).

**Parameters**	**Cell area (μm** ^2^ **)**	**Mean diameter (μm)**	**Perimeter (μm)**	**Circularity**
**Control. Normal**	7.59±1.44^*^	22.10±4.42^*^	61.47±11.74^*^	.026±.005^*^
**Control. High-Fat**	8.23±1.55^**^	24.11±4.83^**^	66.96±12.85^**^	.024±.004^**^
**Normal+1.86 g/kg kiwi**	6.32±1.22‡	19.20±3.76‡	51.97±10.10‡	.030±.006‡
**Normal +3.73 g/kg kiwi**	5.05±0.90^#^	15.33±2.78^#^	41.19±7.52^#^	.038±.006^#^
**High Fat +1.86 g/kg kiwi**	5.45±1.41†	16.45±4.33^#^†	44.72±11.82†	.037±.010^#^†
**High Fat +3.73 g/kg kiwi**	5.60±1.07†	17.11±3.13†	45.85±8.86†	.034±.007†

Means within a column with different symbols (*, **, ‡, ^#^and †) denote significant differences. P<0.05 was accepted as statistically significant. The data are expressed as mean±SD.

**Table 3:** Total phenolic content (TPC) and total flavonoid content (TFC), as well as pyrogallol and vitamin C content of kiwifruit extract

**Type of Extract**	**TPC (mg/g)**	**TFC (mg/g)**	**Pyrogallol (mg/g)**	**Vitamin C(mg/g)**
**Alcoholic fresh**	15.23	0.40	0.303	2.711
**Hydroalcoholic fresh**	12.60	0.76	0.712	0.350
**Alcoholic dried**	14.60	1.19	0.077	3.831
**Hydroalcoholic dried**	11.83	0.51	0.185	0.078

**Total phenolic content and total flavonoid content of kiwifruit**

The total phenolic content and total flavonoid content of various types of kiwi extract are presented in Table 3. 

## Discussion

Exchange of cholesterol ester (CE) between lipoproteins is mediated by CETP (Tall, 1993 ▶). Adipose tissue stores cholesterol and is a major site for lipoprotein interactions (Chung and Parks, 2016). Adipose tissues of human and hamster have high levels of CETP mRNA (Radeauerson et al., 1995; Jiang et al., 1991 ▶). The high-fat diet (HFD) stimulates the CETP synthesis and secretion of adipose tissue (Radeau et al., 1995 ▶). The current research examined whether daily intake of kiwifruit has an effect on adipose tissue histology and CETP gene expression in HFD- fed hamsters or not. In this study, the results of histological examination of adipose tissue showed that the determinant parameters of cell size including cell area, perimeter, diameter, and circularity of cells were larger in HFD-fed hamsters compared to normal diet-fed ones and these parameters decreased significantly in the kiwifruit-receiving groups compared to the control groups. Consistent with our study, it has been reported that taking *Actinidia polygama* for 7 weeks reduced the fat mass and the size of the adipocytes in mice fed with high-fat diet (Sung et al., 2013 ▶). In another study, *A. polygama* extract administration to 3T3-L1 peri-adipocyte cells, inhibited adipocyte differentiation and triglyceride accumulation (Sung et al., 2013 ▶). 

Our investigation revealed that CETP gene expression in adipose tissue increased with kiwifruit supplementation, especially in HFD groups. Also, the present study showed that treatment with kiwifruit reduces cell size parameters such as cell area, mean cell diameter and perimeter in all treated groups compared to their controls. In agreement with this observation, Radeau et al. (1995) ▶ reported that adipose tissue with smaller cells, exhibited higher CETP gene expression. It was reported that CETP in adipose tissue plays a role in cholesterol ester absorption from lipoproteins. It seems that increased expression of CETP in smaller cells from adipose tissue, which has lower lipid content, is required to stimulate the accumulation of more cholesterol in these cells (Radeau et al., 1995 ▶). In the current research, kiwifruit, not only reduced the size of fat cells, but also increased CETP expression. The increases in the CETP expression in kiwifruit-treated groups probably occurred due to a decrease in the cell size of adipocytes. It was reported that in adipose tissue, CETP increases the uptake and accumulation of cholesterol from lipoproteins. This function of CETP may affect the content of intracellular pool of cholesterol in adipose tissues (Radeau et al., 1995 ▶). The accumulation of cholesterol in adipose tissues by CETP may be desirable for the body in two ways. Firstly, cholesterol is stored in adipocyte which is specific for fat storage. Second, cholesterol is absorbed from lipoproteins that results in lower cholesterol concentrations in the plasma and probably less accumulation in other tissues. 

In this study, despite increases in CETP gene expression, the activity of CETP was not changed. Some studies have shown that the use of vitamin E and flavonoid compounds such as xanthohumol leads to a reduction in CETP activity (Hirata et al., 2012 ▶; Shen et al., 1996 ▶). For this reason, in the present study, total phenolic and flavonoid content, pyrogallol and vitamin C was analyzed in kiwifruit extract and were detected in kiwifruit. In the present study, despite the presence of phenolic and flavonoid compounds in kiwifruit extract, CETP activity was not changed in serum of hamsters treated with kiwifruit. Although CETP inhibition has been reported following treatment of vitamin E and flavonoid compounds in *in vitro* studies (Hirata et al., 2012 ▶; Shen et al., 1996 ▶), but these studies were methodologically different from the present study which was carried out *in vivo*. 

 In hamsters, in addition to adipose tissue, CETP is also synthesized in muscle and heart tissues (Jiang et al., 1991 ▶). Therefore, examination of gene expression in these tissues can be helpful in determining whether kiwi directly affects CETP expression or not.

Various studies have shown that the use of compounds such as vitamins E and C, quercetin, and polyphenols leads to increases in HDL-C levels (Shen et al., 1996 ▶; JuŸwiak et al., 2005 ▶; Recio-Rodriguez et al., 2015 ▶). Although the reduction of CETP activity in serum can lead to an increase in HDL-C, other factors can also increase HDL-C concentration. In this study, serum CETP activity in the kiwifruit-treated groups did not differ from the untreated groups, so increasing the amount of HDL-C can be due to the effects of other factors. In the present study, consumption of kiwifruit (3.73 g/kg) significantly increased the HDL-C level in the HFD-fed group. Phenol compounds, flavonoids and vitamins such as vitamins E and C of kiwi have an important role in enhancement of HDL-C levels. It has also been shown that taking kiwifruit increases the protein level of Apo A1, which forms the major structural component of HDL (Recio-Rodriguez et al., 2015 ▶). Given the helpful effects of kiwifruit, this fruit should be eaten over longer periods of time to yield constant effects. According to the results of this study, the use of kiwifruit as an available fruit can help to prevent the onset of dyslipidemia and related diseases in healthy people. Kiwifruit in people with hyperlipidemia, along with chemical drugs, may accelerate the treatment process.

Kiwifruit consumption reduces the size of adipocytes and increases CETP gene expression in these cells. Despite increased CETP expression in adipose tissue, its activity in serum was not changed following kiwifruit supplementation.
